# Occupational exposure to pesticides and health symptoms among family farmers in Brazil

**DOI:** 10.11606/s1518-8787.2020054002263

**Published:** 2020-11-27

**Authors:** Rafael Junqueira Buralli, Helena Ribeiro, Verónica Iglesias, María Teresa Muñoz-Quezada, Renata Spolti Leão, Rejane Correa Marques, Milena Maria Cordeiro de Almeida, Jean Remy Davée Guimarães

**Affiliations:** I Universidade de São Paulo Faculdade de Saúde Pública Programa de Pós-Graduação em Saúde Pública São PauloSP Brasil Universidade de São Paulo. Faculdade de Saúde Pública. Programa de Pós-Graduação em Saúde Pública. São Paulo, SP, Brasil; II Universidade de São Paulo Faculdade de Saúde Pública Departamento de Saúde Ambiental São PauloSP Brasil Universidade de São Paulo. Faculdade de Saúde Pública. Departamento de Saúde Ambiental. São Paulo, SP, Brasil; III Universidad de Chile Escuela de Salud Pública Departamento de Epidemiología Santiago Chile Universidad de Chile. Escuela de Salud Pública. Departamento de Epidemiología. Santiago, Chile; IV Universidad Católica del Maule Facultad de Ciencias de la Salud TalcaMaule Chile Universidad Católica del Maule. Facultad de Ciencias de la Salud. Talca, Maule, Chile; V Universidade Federal de Minas Gerais Centro de Tecnologia em Nanomateriais e Grafeno Belo HorizonteMG Brasil Universidade Federal de Minas Gerais. Centro de Tecnologia em Nanomateriais e Grafeno. Belo Horizonte, MG, Brasil; VI Universidade Federal do Rio de Janeiro Centro Multidisciplinar MacaéRJ Brasil Universidade Federal do Rio de Janeiro, Campus Macaé. Centro Multidisciplinar - UFRJ. Macaé, RJ, Brasil; VII Universidade Federal da Bahia Instituto de Ciências da Saúde Departamento de Fisioterapia SalvadorBA Brasil Universidade Federal da Bahia. Instituto de Ciências da Saúde. Departamento de Fisioterapia. Salvador, BA, Brasil; VIII Universidade Federal do Rio de Janeiro Instituto de Biofísica Carlos Chagas Filho Rio de JaneiroRJ Brasil Universidade Federal do Rio de Janeiro. Instituto de Biofísica Carlos Chagas Filho. Rio de Janeiro, RJ, Brasil

**Keywords:** Farmers, Pesticide Exposure, Mental Disorders, epidemiology, Occupational Health

## Abstract

**OBJECTIVE:**

To explore the association of occupational pesticide exposure with acute and mental health symptoms.

**METHODS:**

Cross-sectional survey carried out with 78 Brazilian family farmers, who were pesticide applicators and helpers conveniently selected. Symptoms and exposure data were collected by interviews, and mental health outcomes by the Self-Reporting Questionnaire. Blood samples were analyzed to assess cholinesterase levels. Exposure indicators and symptoms were compared between applicators and helpers, and Poisson regression was performed to estimate prevalence ratios.

**RESULTS:**

Farmers reported exposure to multiple pesticides from early ages; they worked without safety training, technical support, and full protective equipment, and they had a high prevalence of acute and mental health symptoms (e.g., headache, mucosal irritation, tachycardia, and depressive signs). Applicators had more cholinesterase changes than helpers, but less symptoms. Helpers used less personal protection and had significantly higher prevalence ratio of headache, dyspnea, wheezing, cough, poor digestion, tiredness, and feeling worthless, after adjustment.

**CONCLUSIONS:**

Acute and mental health symptoms were observed, both among farmers and helpers. Thus, surveillance actions must be reinforced in Brazil, technical support and safety training improved, focused on applicators and helpers, who are occupationally and environmentally exposed to pesticides. Agricultural practices of these groups with less pesticide use should receive incentive.

## INTRODUCTION

Excessive and unsafe use of pesticides represents a serious risk to human health, environment, and quality of food. About 25 million people experience unintentional pesticide poisoning yearly worldwide ^1^ , resulting in 200,000 deaths, mainly affecting low- and middle-income countries (LMIC) ^2^ . In LMIC, the occupational exposure to pesticides has been associated with gastrointestinal, musculoskeletal, respiratory, allergic, and nervous effects ^3^^–^^6^ , and common mental disorders (CMD) such as depression, anxiety, and suicide ^7^^–^^9^ . However, these adverse effects are not restricted to LMIC, and occupational exposure to pesticides was associated with health outcomes in high-income countries such as the USA, England, South Korea, and Spain ^10^^,^^11^ .

Between 2010 and 2015, more than 600,000 pesticide poisoning cases and 2,074 deaths occurred in Brazil ^12^ , but the cases are vastly underreported by national information systems. It is estimated that for every registered case there are 50 unregistered ones ^13^ . Mental illness is a major public health concern in terms of lost health and burden of disease, and its symptoms are often overlooked by health services ^14^ . Depression and anxiety affect, respectively, 5.8% and 9.3% of the Brazilian population, more than 4.4% and 3.6% affected worldwide ^15^ .

Farmers from LMIC, mostly located in tropical areas with easy pest proliferation, tend to be more exposed to pesticides due to the lack of safety regulation, surveillance and training, increased use of highly toxic chemicals, low risk awareness, misuse of personal protective equipment (PPE), and careless handling and pulverization ^2^^,^^4^^,^^5^ . Studies conducted in Brazil showed that farmers commonly use complex mixtures of pesticides without precautionary measures, which could potentially reduce exposure and protect their health ^16^^–^^18^ .

Brazil is a world's leading agricultural producer, and the largest consumer of pesticides since 2008, trading highly toxic chemicals banned in many countries ^13^ . Cholinesterase-inhibitor pesticides, such as organophosphates (OP) and carbamates (CM), represent an important risk for human health and are considered the main responsible for pesticide poisoning in LMIC ^4^ .

Family farming is the primary income source for 40% of the active population in Brazil and for 90% of municipalities with fewer than 20,000 inhabitants ^19^ .

Although studies conducted in Brazil had explored the health effect of pesticide use ^8^^,^^9^^,^^18^^,^^20^ , many regions and crops are still underrepresented, and mental health and tomato growers were not studied recently. Therefore, this study aims to explore the association between pesticide exposure and the prevalence of self-reported acute and mental health symptoms among family farmers in São José de Ubá, state of Rio de Janeiro.

## METHODS

### Study Area and Sample

This cross-sectional study was conducted in July and August 2014, at the end of the crop season, in São José de Ubá (SJU), located in a mountainous region in the Northwest of the state of Rio de Janeiro, Brazil ( Figure ). SJU is a small municipality of 7,000 inhabitants, where 55% live in the rural area, 16% has formal employment, and 40% have a monthly per capita income equivalent to $100 US dollars or less than half the national minimum wage ^19^ . SJU is one of the largest tomato producers in Brazil, and the municipality income depends on smallholder family farming ^19^ . Tomato cultivation demands an intensive care for pest control, commonly based on the use of large amounts of pesticides ^13^ . Between 2007 and 2017, the yearly tomato production in SJU ranged between 21,000 and 32,000 tons ^19^ . Previous studies revealed soil degradation and water contamination as a consequence of intensive farming and livestock practices ^21^ .

**Figure:**
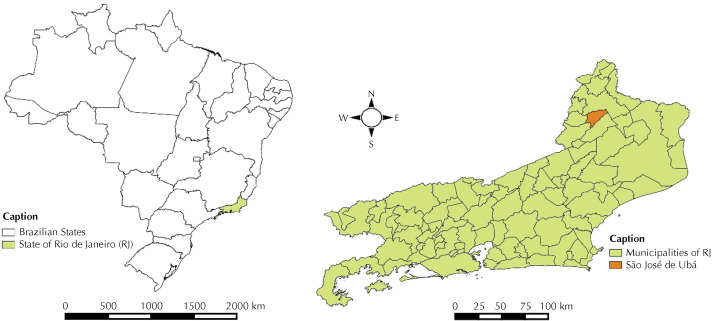
Map of study location, *São José de Ubá* , State of Rio de Janeiro, Brazil.

The study sample was composed of 78 family farmers, older than 18 years, recruited sequentially by convenience in agricultural areas of SJU with word-of-mouth of residents and stakeholders (snowball sampling). Almost all (95%) individuals recruited agreed to participate in the study, and participants represented about 11% of tomato growers in SJU. The final sample size was delimited by the time and budget constraints of the project.

This study is part of a broader project assessing human health risk by pesticides and metals exposure in SJU, and the study methodology ^21^ and pulmonary function impairment ^22^ were discussed elsewhere. The study protocol was approved by the Ethical Board of the *Hospital Universitário Clementino Fraga Filho* of the *Universidade Federal do Rio de Janeiro* , and all participants provided written informed consent. Test results were delivered individually, and guidance on health protection provided. Individuals with significant outcomes were referred to the municipal healthcare service.

Based on observational visits and self-declared cultivation tasks, participants (n=78) were divided into two groups as a proxy of exposure: a) applicators (n=42), who were daily involved in all crop activities, including pesticide handling and spraying; and b) current or former helpers (n=36), who were farmer's relatives performing additional crop-related tasks, but not pesticide spraying.

Individual interviews based on questionnaire were carried out at participant's households, neighborhood schools and healthcare units to ease community engagement and assimilate the local culture. Socioeconomic and demographic data such as age, gender, marital status, body mass index (BMI), educational level, smoking habits, and alcohol consumption were obtained. Monthly family income was estimated based on the Brazilian minimum wage (R$ 998 in 2019), which was equivalent to approximately US$ 240 and now is around US$ 200.

### Exposure Assessment

Participants were asked about their current and previous pesticide exposure, including: duration of work with pesticides, age of first exposure working or helping at crops, home distance from crop areas, use of recommended PPE (cloth mask, visor, hat, gloves, boots, and overall), types of pesticide frequently used, previous training and technical support received, residential exposure to pesticides (either by use for pest control or by contact with contaminated clothes and equipment), and poisoning history. Safety practices (e.g. eating at the field, washing hands, and showering after crop activities) were also investigated.

Some farmers refused to participate in blood tests or had insufficient samples. Samples from 70 participants were collected by qualified health personnel to evaluate acetylcholinesterase (AChE) and butyrylcholinesterase (BChE) inhibition, which are respectively biomarkers of chronic and acute exposure to OP and CM pesticides ^13^ . The AChE and BChE biomarkers are useful for an initial screening of pesticide poisoning, although they have low specificity and sensitivity, and they are not effective to assess other chemical classes of pesticides ^17^ .

Measurements were performed by the Centro de Estudos da Saúde do Trabalhador e Ecologia Humana (CESTEH/FIOCRUZ) by the modified Ellman method ^23^ , in which reference values were defined as 0.56 mmol/min/mg for AChE for both genders, and 2.29 for BChE for men and 1.61 mmol/min/mg for women. Values above these were considered normal.

### Self-Reported Symptoms and Mental Health Assessment

Twenty-three acute symptoms previously associated with pesticide poisoning were presented to the participants, who were asked to confirm whether they regularly had them or not. Prevalence of probable CMD was assessed by the Self-Reporting Questionnaire (SRQ-20), proposed by WHO as a low-cost and easy tool for psychiatric screening, and it is recommended for community studies and basic care. The questionnaire has 20 binaries questions (yes/no) about depressive and anxiety signs, reduced vital energy, and somatic symptoms. It was validated in Brazil with good internal consistency ^24^ , high sensitivity (83%) and specificity (80%) ^25^ , and the standard cutoff was set as six or more positive answers for men and eight or more for women ^14^ .

### Statistical Analysis

The prevalence of sociodemographic and pesticide exposure data was compared between occupational groups (pesticide applicators *versus* helpers). Data with normal distribution were presented as mean and standard deviation (SD), and with non-normal distribution as median and interquartile range (IQR 25-75%). Pearson's chi-square test was used to compare categorical variables, and Fisher's exact test was used for variables with less than five observations. Continuous variables with normal distribution were compared among groups by T-test, and with non-normal distribution by Rank-sum. Cholinesterase activity was compared with the reference values and among groups ^23^ . The prevalence of each acute and mental health symptom was compared between applicators and helpers, and possible associations tested by Poisson regression with robust variance, adjusted by potential confounders, namely age, BMI, smoking habit, gender, and alcohol consumption. Family income was similar among groups; thus it was not included in the analysis. Statistical analysis was performed using Stata 14 (Stata Corp., College Station, TX, USA) and SPSS 23 (IBM Corp., Chicago, IL, USA). *p* -values < 0.05 were considered significant.

## RESULTS

Most participants were married, had low educational level and family income, they had never smoked or were former smokers, and did not consume alcohol. Most applicators were men (83.3%), had a mean age of 40.3 years, and worked with crops for 27 years. Current or former helpers were mostly women (72.2%), had a mean age of 48.4 years, worked or helped in crop activities for 27 years, and had a significantly higher BMI ( Table 1 ).

**Table 1:** Sociodemographic characteristics among family farmers in São José de Ubá, Brazil, divided by occupational groups. Crop season, 2014.

Sociodemographic characteristics		Total % (n = 78)	Applicators % (n = 42)	Current or former helpers % (n = 36)	p
Age (mean in years; SD)		44.1; 13.2	40.3; 11.3	48.4; 14.1	0.08 c
Gender					
	Male	57.7 (45)	83.3 (35)	27.8 (10)	< 0.001 d
	Female	42.3 (33)	16.7 (7)	72.2 (26)	
Marital status					
	Single or divorced	14.1 (11)	14.3 (6)	13.9 (5)	0.96 d
	Married/cohabiting partner	85.9 (67)	85.7 (36)	86.1 (31)	
Monthly family income a					
	Up to 2 wages	71.8 (56)	78.6 (33)	63.9 (23)	0.15 d
	More than 2 wages	28.2 (22)	21.4 (9)	36.1 (13)	
Body Mass Index b		25.7 (22.4–28.5)	24.5 (20.8–27.9)	26.5 (23.4–31.9)	0.03 e
	Low or normal weight	46.7 (35)	55.0 (22)	37.1 (13)	0.05 f
	Overweight	36.0 (27)	37.5 (15)	34.3 (12)	
	Obese	17.3 (13)	7.5 (3)	28.6 (10)	
Years of education b		4.0 (3.0–8.0)	4.0 (3.5–8.0)	4.5 (3.0–7.8)	0.71 e
Smoking status					
	Never	66.7 (52)	66.7 (28)	66.7 (24)	0.46 f
	Former	20.5 (16)	16.7 (7)	25.0 (9)	
	Current	12.8 (10)	16.7 (7)	8.3 (3)	
	Mean/Median (pack-years)	0 (0–2)	4.7 / 0.0	4.2 / 0.0	0.86 e
Alcohol consumption (if yes)		29.5 (23)	33.3 (14)	25.0 (9)	0.42 d

a Brazilian minimum wage was used as basis, which in 2019 was R$ 998 (~ 240 US$).

b Data with non-normal distribution shown as median and interquartile range (IQR).

c T-test.

d Pearson's chi-square test.

e Rank-sum.

f Fisher's exact test.

Most participants started to help in crop activities at early ages, lived up to 1km from crop areas, never had technical support or safety training to work with pesticides, and they were domestically exposed to pesticides by using them for household pest control or having contact with contaminated clothes or equipment. Most applicators directly handled or sprayed pesticides for 1–3 days (85.7%) or 4–7 days (11.9%) per week during the crop season, while helpers never or rarely handled pesticides. Applicators affirmed to use significantly more PPE than helpers (although not complete), and considered visors hot and uncomfortable, thus they did not use them often ( Table 2 ).

**Table 2:** Pesticide exposure characteristics among family farmers in São José de Ubá, Brazil, divided by occupational groups. Crop season, 2014.

Exposure characteristics		Total % (n = 78)	Applicators % (n = 42)	Current or former helpers % (n = 36)	p
Years of work with crops (mean ± SD)		27.0 ± 14.3	27.0 ± 12.4	27.1 ± 16.4	0.92 d
Age of first exposure at crop					
	Up to 12 years old	50.0 (39)	59.5 (25)	38.9 (14)	0.08 e
	Between 13-17 years old	29.5 (23)	28.6 (12)	30.6 (11)	
	More than 18 years old	20.5 (16)	11.9 (5)	30.6 (11)	
Home exposure (if yes)		87.2 (68)	81.0 (34)	94.4 (34)	0.08 e
Residential distance from crop					
	Up to 1km	84.6 (66)	85.7 (36)	83.3 (30)	0.77 e
	More than 1 km	15.4 (12)	14.3 (6)	16.7 (6)	
Use of PPE a					
	Use of any PPE	67.9 (53)	95.2 (40)	36.1 (13)	<0.001 e
	Use of cloth mask	50.0 (39)	78.6 (33)	16.7 (6)	<0.001 e
	Use of visor	14.1 (11)	21.4 (9)	5.6 (2)	0.06 f
	Use of hat	37.2 (29)	59.5 (25)	11.1 (4)	<0.001 f
	Use of gloves	52.6 (41)	76.2 (32)	25.0 (9)	<0.001 e
	Use of boots	53.8 (42)	76.2 (32)	27.8 (10)	<0.001 e
	Use of overcoat	39.7 (31)	59.5 (25)	16.7 (6)	<0.001 e
Previous poisoning (if yes)		17.9 (14)	16.7 (7)	19.4 (7)	0.75 e
Received training/technical support		14.1 (11)	19.0 (8)	8.3 (3)	0.21 f
Washes hands after working at crop		80.8 (63)	88.1 (37)	72.2 (26)	0.08 e
Takes shower after working at crop		60.3 (47)	69.0 (29)	50.0 (18)	0.09 e
Consumes food and water in the field		91.0 (71)	95.2 (40)	86.1 (31)	0.16 e
Cholinesterase tests b					
	AChE (mean ± SD)	1.29 ± 0.42	1.23 ± 0.44	1.35 ± 0.38	0.25 d
	BChE (mean ± SD)	3.30 ± 1.06	3.16 ± 1.09	3.48 ± 1.02	0.21 d
	BChE below the RV c	20.0 (14)	28.2 (11)	9.7 (3)	0.07 f

a PPE: Personal protective equipment

b *n* = 70 samples, and values are expressed in mmol/min/mg

c RV: reference values, for AChE = 0.56 (for both genders), and BChE = 2.29 mmol/min/mg for men and 1.61 mmol/min/mg for women

d T-test

e Pearson's chi-square test

f Fisher's exact test.

All participants had normal AChE values (above the reference level), while 11 applicators (28%) and three helpers (10%) presented inhibited BChE (below the reference level), without significant differences between groups. Applicators presented lower mean values for AChE and BChE, but they were not statistically significant ( Table 2 ).

Regarding the products used, 49 pesticides from 31 chemical groups were cited, mainly OP pesticides such as Acephate and Chlorpyrifos, CM pesticides such as Mancozeb and Methomyl, pyrethroids such as Lambda-Cyhalothrin and Deltamethrin, and also nitriles, diamides, neonicotinoids, avermectins, and benzimidazole. Most of these chemicals are classified as extremely and highly toxic to humans ^26^ . About 30% of applicators did not know which pesticides they were applying because someone else does the mixture and they only apply. Glyphosate and Paraquat were cited by 35% and 17%, respectively, but they are prohibited for tomato cultivation in Brazil ^13^ . The extremely toxics Chlorpyrifos and 2,4-D are forbidden for tomato crops in Brazil, and the highly toxic Endosulfan was already banned, but it was also mentioned.

Overall, only 11.5% of participants did not report any acute symptom, while 27% had between one and three, 45% between four and nine, and 16.7% more than 10 symptoms (out of 23). The symptoms most commonly reported were: mucosal irritation (41%), headache (40%), tachycardia (36%), lower limbs fatigue and palpitation (33%), dizziness and blurred vision (29%), stomach pain (28%), and cramps (27%) ( Table 3 ).

**Table 3:** Prevalence of self-reported acute symptoms and prevalence ratio (PR) of occupational group during the crop-season (2014) in São José de Ubá, Brazil.

Acute symptoms	Applicators % (n = 42)	Helpers % (n = 36)	Crude model		Adjusted model b	
			PR a	95%CI	PR a	95%CI
Headache	31.0 (13)	50.0 (18)	1.62	0.92–2.83	2.09	1.09–4.01 d
Dizziness	26.2 (11)	33.3 (12)	1.27	0.64–2.54	0.83	0.35–2.01
Tremors	7.1 (3)	22.2 (8)	3.11	0.88–10.95	2.69	0.73–9.94
Tingling in upper limbs	7.1 (3)	38.9 (14)	5.44	1.69–17.58 d	3.12	0.77–12.66
Tingling in lower limbs	4.8 (2)	33.3 (12)	7.00	1.66–29.50 d	3.33	0.57–19.28
Muscle weakness	16.7 (7)	27.8 (10)	1.67	0.70–3.95	0.70	0.24–2.04
Lower limbs fatigue	21.4 (9)	47.2 (17)	2.20	1.12–4.34 d	1.05	0.48–2.26
Blurred vision	31.0 (13)	27.8 (10)	0.90	0.45–1.80	0.83	0.31–2.20
Photophobia	14.3 (6)	30.6 (11)	2.14	0.87–5.24	2.47	0.94–6.46
Cramps	16.7 (7)	38.9 (14)	2.33	1.05–5.17 d	1.71	0.58–5.06
Tinnitus	21.4 (9)	22.2 (8)	1.04	0.44–2.41	1.22	0.51–2.93
Excessive salivation	14.3 (6)	11.1 (4)	0.78	0.24–2.56	0.95	0.29–3.19
Nausea/vomiting	19.0 (8)	19.4 (7)	1.02	0.41–2.55	0.99	0.35–2.76
Lack of appetite	7.1 (3)	16.7 (6)	2.33	0.62–8.74	1.06	0.26–4.38
Stomach pain	19.0 (8)	38.9 (14)	2.04	0.96–4.33	1.13	0.51–2.51
Skin irritation	16.7 (7)	25.0 (9)	1.50	0.62–3.64	0.52	0.18–1.49
Mucosal irritation	38.1 (16)	44.4 (16)	1.17	0.68–1.99	0.80	0.41–1.55
Tachycardia	38.1 (16)	33.3 (12)	0.88	0.48–1.60	0.70	0.32–1.55
Palpitation	28.6 (12)	38.9 (14)	1.36	0.72–2.56	0.91	0.39–2.11
Excessive sweating	9.5 (4)	13.9 (5)	1.46	0.42–5.07	0.83	0.17–4.15
Dyspnea	11.9 (5)	38.9 (14)	3.27	1.30–8.24 d	3.83	1.54–9.52 d
Wheezing	2.4 (1)	27.8 (10)	11.67	1.55–87.93 d	16.07	2.37–108.75 d
Cough	16.7 (7)	30.6 (11)	1.83	0.79–4.26	2.64	1.07–6.50 d
Total of symptoms c	3.5 (1.0-6.0)	6.5 (3.3-10.5)	1.70	1.19–2.42 d	1.32	0.86–2.03

a Prevalence ratio: helpers *versus* applicators as reference group

b Adjusted by age, BMI, and smoking habits (pack-years), all continuous variables, and gender (male = 1, female = 2) and alcohol consumption (yes/no)

c Sum of symptoms reported by each subject, presented as median and IQR – interquartile range (25–75%)

d Data with significant p-Value (≤ 0.05).

Generally, symptoms were more prevalent among helpers than applicators, except for blurred vision, excessive salivation, and tachycardia. Significant differences among occupational groups were found for tingling in upper and lower limbs, fatigue, cramps, dyspnea, wheezing, and total of acute symptoms, all with higher prevalence ratios among helpers. After adjusted for potential confounders, helpers showed significantly higher prevalence ratios than applicators for headache (PR = 2.09; 95%CI 1.09–4.01), dyspnea (PR = 3.83; 95%CI 1.54–9.52), wheezing (PR = 16.07; 95%CI 2.37–108.75), and cough (PR = 2.64; 95%CI 1.07–6.50). Moreover, a prevalence higher than 30% was observed for dizziness, photophobia, stomach pain, palpitation, and cough among helpers, and for headache, mucosal irritation, and tachycardia in both groups, although without significant differences between groups ( Table 3 ).

Helpers showed higher prevalence of all mental health symptoms in the SRQ-20, except for daily work suffering. Statistically significant differences in PR were observed between occupational groups for poor sleep, feeling easily tired, unable to play a useful part, and feeling worthless, all with higher PR among helpers, while daily work suffering was higher among applicators. After adjustment for age, BMI, smoking habit, gender and alcohol consumption, higher PR among helpers was observed for poor digestion (PR = 7.85; 95%CI 1.17–52.89), feeling easily tired (PR = 3.20; 95%CI 1.33–7.66), and feeling worthless (PR = 7.23; 95%CI 1.69–31.04). Furthermore, some mental health symptoms had a prevalence as high as 50% or 30%, but without significant differences between groups ( Table 4 ).

**Table 4:** Prevalence of affirmative answers of the Self-Reporting Questionnaire (SRQ-20), probable common mental disorder, and prevalence ratio (PR) among smallholder family farmers, divided by occupational group during the crop-season (2014) in São José de Ubá, Brazil.

SRQ-20 – Affirmative answers		Prevalence (Crude model)				Adjusted model b	
		Applicators % (n = 42)	Helpers % (n = 36)	PR a	95%CI	PR a	95%CI
Depressive/Anxious signs							
	Feel nervous, tense or worried	54.8 (23)	69.4 (25)	1.27	0.89–1.80	1.28	0.85–1.93
	Easily frightened	33.3 (14)	50.0 (18)	1.50	0.87–2.58	1.74	0.99–3.07
	Feel unhappy	14.3 (6)	30.6 (11)	2.14	0.87–5.24	1.76	0.58–5.31
	Cry more than usual	9.5 (4)	25.0 (9)	2.63	0.88–7.87	1.90	0.62–5.76
Somatic symptoms							
	Often have headaches	28.6 (12)	38.9 (14)	1.36	0.72–2.56	1.53	0.69–3.42
	Poor sleep	26.2 (11)	55.6 (20)	2.12	1.18–3.82 d	1.08	0.53–2.23
	Uncomfortable stomach feelings	21.4 (9)	27.8 (10)	1.30	0.59–2.85	0.99	0.41–2.35
	Poor digestion	2.4 (1)	16.7 (6)	7.00	0.87–56.20	7.85	1.17–52.89 d
	Poor appetite	9.5 (4)	22.2 (8)	2.33	0.76–7.16	1.11	0.37–3.34
	Hands shake	21.4 (9)	25.0 (9)	1.17	0.52–2.64	1.14	0.43–2.99
Reduced vital energy							
	Feeling easily tired	16.7 (7)	47.2 (17)	2.83	1.32–6.08 d	3.20	1.33–7.66 d
	Difficulty in making decisions	21.4 (9)	30.6 (11)	1.43	0.66–3.06	0.69	0.27–1.79
	Difficulty in enjoying daily activities	11.9 (5)	25.0 (9)	2.10	0.77–5.74	1.11	0.38–3.29
	Daily work suffering	38.1 (16)	16.7 (6)	0.44	0.19–1.00 d	0.48	0.21–1.10
	Feeling tired all the time	19.0 (8)	36.1 (13)	1.90	0.88–4.07	1.86	0.76–4.54
	Trouble thinking clearly	21.4 (9)	33.3 (12)	1.56	0.74–3.28	1.18	0.56–2.50
Depressive thoughts							
	Unable to play a useful part	7.1 (3)	25.0 (9)	3.50	1.02–12.05 d	2.35	0.68–8.10
	Lost interest in things	11.9 (5)	25.0 (9)	2.10	0.77–5.74	1.54	0.43–5.54
	Thought of ending your life	7.1 (3)	16.7 (6)	2.33	0.62–8.74	1.60	0.41–6.15
	Feeling worthless	4.8 (2)	36.1 (13)	7.58	1.82–31.68 d	7.23	1.69–31.04 d
Probable CMD c		23.8 (10)	44.4 (16)	1.87	0.97–3.60	1.85	0.92–3.72

a Prevalence ratio: helpers *versus* applicators as reference group

b Adjusted by age, BMI, and smoking habits (pack-years), all continuous variables, and gender (male = 1, female = 2) and alcohol consumption (yes/no)

c Probable common mental disorder (CMD) = individuals above the cutoff level (six or more positive answers for men and eight or more for women)

d Data with significant p-value (≤ 0.05).

## DISCUSSION

Our study shows that smallholder family farmers in SJU were occupationally and environmentally exposed to pesticides from an early age, lived near crops, worked without safety training, technical support and full recommended PPE, and had a considerable number of acute and mental health symptoms. First, our hypothesis was that applicators are more exposed to pesticides and have more symptoms than helpers who assist in crop activities; however, most symptoms had higher prevalence among current and former helpers, even after adjusting for possible confounders, including gender. An explanation may be that helpers are also very exposed to pesticides due to their higher residential exposure, lower training, and involvement in re-entry tasks on the same day or day after spraying using less PPE. Selection bias could have occurred when considering the farmers who stopped working because they felt ill as former helpers, though it could also be due to residual uncontrolled confounders. Compared with helpers, participants in the pesticide applicators group were mostly younger men who started to work in agriculture at an earlier age and handled multiple pesticides about three times a week, had a higher duration of exposure at crops, but lower residential exposure.

The symptomatology presented in this study must be interpreted with caution due to the small sample size, although our findings are supported by previous studies conducted in other LMIC, and also high-income countries. In our study, farmworkers performing re-entry activities were less trained, used fewer PPE, performed less hygienic practices, and showed more symptoms than pesticide applicators from Ethiopia ^6^ and Chile ^4^ .

Moreover, participants in our study had less safety guidance, used less protection and had higher prevalence of symptoms than both pesticide applicators and non-applicators from Zanzibar ^27^ . On the other hand, coffee farmers from Dominican Republic exposed to multiple pesticides without using PPE (e.g., only 13% used masks and gloves) had a higher prevalence of all symptoms than participants in our study ^5^ . These findings emphasize the role of protection equipment and technical support in poisoning prevention.

High-income countries have overcome the acute pesticide poisonings and are more concerned about chronic effects and long-term exposure. But acute and chronic poisonings still persist and are growing problems for LMIC ^6^ . According to the WHO, an acute pesticide poisoning must present clear signs of exposure, temporal cause-effect relationship, and at least three symptoms compatible with the exposure ^28^ . In our study, participants were asked to confirm which symptoms they regularly have, so we cannot ensure temporality; however, most individuals were continuously exposed, and 60% of them had more than four acute symptoms suggestive of pesticide poisoning.

Regarding mental health, helpers in SJU had higher prevalence than applicators in all questions about depressive and anxiety symptoms, and nearly twice as high CMD prevalence (44% *versus* 24%). Other studies assessed the CMD of Brazilian farmers with SRQ-20, and results vary widely. A study found a CMD prevalence of 34%, being significantly higher for women (40%) than men (26%) ^7^ . Another study with tobacco farmers found a CMD prevalence of 12%. Although this prevalence is much lower than in our study, they observed a higher PR for women (PR = 1.39; 95%CI 1.12–1.72), and individuals performing re-entry tasks (PR = 1.71; 95%CI 1.33–2.20) ^8^ , which is consistent with our study. Another study with family farmers in Brazil reported a CMD prevalence of 27% for both genders, which is higher than for applicators but lower than for helpers in our study ^9^ .

A review found that a higher prevalence of mental health outcomes, notably depression, anxiety, and suicide attempts, were found positively associated with exposure to pesticide among farmers in high-income countries as the USA, England, South Korea, and Spain ^10^ . Depression was significantly associated with a history of pesticide poisoning, but not low or high cumulative exposure in spouses of pesticide applicators in the USA ^29^ .

The high variability of SRQ-20 can be explained by the questionnaire high sensitivity and capacity to identify a large spectrum of affections ^9^ , but also by biological and social factors (e.g. women tend to report more symptoms, and less educated individuals tend to over-report mental health complaints) ^14^ .

Previous studies conducted with Brazilian farmers indicated a scenario of exposure similar to our findings: low educational level and income, residential proximity to crop areas, working in crops since childhood, poor technical support and safety training, and lack or misuse of PPE ^16^^,^^18^ . Inadequate safety practices (e.g. drinking and eating at field and showering only at the end of workday) may increase pesticide exposure ^3^^,^^4^ . Moreover, poor understanding of pesticide instruction leaflets, and influence of neighbors on pesticide user's decisions, especially regarding handling and dosage, may compromise farmer's ability to reduce exposure and protect their health ^16^ .

In Brazil, complex mixtures of multiple pesticides are commonly sprayed by manual pumping or backpack tanks by smallholder family farmers ^16^^,^^17^ , which may result in potential additive and synergic effects, and greater health outcomes ^1^^,^^13^ . Pesticides are authorized for specific target-crops in Brazil, and the use of prohibited chemicals or those not allowed for tomato crops was observed in our study. Some pesticides banned or sale-restricted in other countries due to their high toxicity, such as Abamectin, Acephate, Glyphosate, and Paraquat, are still highly commercialized in Brazil ^13^ , and their use was mentioned in our study. Most symptoms found in our study were previously associated with OP pesticides exposure ^11^ , but the effects of pesticide mixtures cannot be ruled out and deserve further attention.

Major challenges on evaluating health effects of pesticide exposure among family farmers in Brazil are their widespread distribution, their continuous exposure to multiple chemicals, large distance of crop areas to health services, and the shortage of laboratories with analytical capacity ^13^^,^^17^ . According to the Brazilian law, all farmers must be periodically subjected to medical exams and cholinesterase tests, albeit this is not provided to millions of smallholder family farmers distributed in 4.4 million properties ^19^ . Cholinesterase enzymes are useful for screening of OP and CM poisoning or continuous monitoring, despite their high variability, and low sensitivity and specificity ^17^ . Measurements of urinary biomarkers are more suitable exposure assessment ^11^ , though expensive and seldom available in Brazil. In our study, applicators had more BChE inhibition and lower cholinesterase levels, which may indicate continuous exposures to high doses, avoiding a complete BChE recovery, but it contrasts with the fact that more symptoms had higher prevalence among helpers.

Another study with Brazilian tomato farmers showed that 62% reported more than one illness after pesticide exposure, but only 21% of poisoned workers sought a health service and 70% self-medicated ^20^ . This low demand for health services, especially in less severe cases, compromises the visibility and hinders the understanding of the real dimension of the problem ^13^ .

The lack of an unexposed control group and the cross-sectional design were some limitations of this study. A longitudinal study with more sensible biomarkers would enable the evaluation of health effects over time and precisely associate them with exposure. Moreover, the convenience sampling and sample size of our study limit external validity. Thus, this study assumes an exploratory purpose, which only allows the assessment of associations but not cause-effect relationships, although indicating relevant hypotheses to be further investigated. Possible information and memory bias were minimized by an experienced health professional conducting the interviews, the access to recent symptoms, and trust relationships with participants. Moreover, our questionnaire was based on validated protocols, and pilot interviews ensured that the questionnaire was easily understood.

This investigation contributes to filling the data gap concerning occupational exposure to pesticides and health effects in Brazil by providing complementary evidence about family farmers, responsible for about 70% of food (mostly vegetables) consumed in the country ^13^ . This study highlights the demand for a more efficient technical support stimulating farmers to use chemicals consciously and better protect themselves. The strengthening of public policies that address the current vulnerability and risk of family farmers is urgent. Moreover, we strongly recommend a careful overhaul of the Brazilian legislation to restrict hazardous pesticides and encourage agricultural practices less dependent on pesticides for family farmers.

## CONCLUSIONS

Our study indicates that Brazilian family farmers work without proper technical support and safety behavior that could reduce pesticide exposure and protect their health. Also, it highlights that public policies must target not only the applicators, but also other workers who often assist in crop activities and vulnerable groups both occupationally and environmentally exposed. The high exposure to pesticides must be a major public health concern because it reduces farmers' quality of life, affects the rural workforce, increases morbidity and mortality burden of diseases and health costs. Thus, we strongly recommend the strengthening of surveillance actions, technical support, and educational programs aiming at family farmers exposed to pesticides in Brazil. Promoting sustainable agriculture is the most effective way to protect farmers' health, general population, and the environment.
